# Supplementary material for: Time efficiency as a mediator between institutional support and higher education student engagement during e-learning
Source: PLoS One. 2025 Jan 24;20(1):e0315420. doi: 10.1371/journal.pone.0315420 (PMC11760031; doi:10.1371/journal.pone.0315420)
Supplement: Attachment — Submitted filename: Response-to-rev-comments.docx [file pone.0315420.s001.docx]

**Dear Reviewers,**

We sincerely appreciate your very helpful feedback on our draft. We have carefully incoporated your comments and made significant revisions to improve the clarity, organization, and quality of the paper. Below, we list how we have addressed your concerns.

**Editor**

We have included the appropriate statement regarding the funder's role in the cover letter. Additionally, we have submitted the data as a supplementary file.

**Reviewer 1**

1. Abstract

We updated the first sentence to make it clearer and easier to understand. We eliminated the term *questionnaire-based* as advised.

2. Introduction

We updated the statement regarding e-learning to better reflect its historical context and recent advances. We increased sentence linking to improve cohesiveness and comprehension. We spelled out UNESCO in full at the first mention. We improved the clarity of the second paragraph by clarifying the grammar. We have plainly stated the gap in knowledge and its significance (as a closing paragraph of the literature review).

3. Literature Review

We changed the first statement to add examples of fields where time efficiency has been explored. We have presented an enhanced argument for time-efficiency's mediation role. We have updated the abbreviation format for Engagement Theory (ET) and eliminated use of acronym ET. We proofread and edited the manuscript, correcting punctuation, paragraph length, and redundancy. We have expanded the concept of Engagement Theory to better support its application in our study. We adjusted the linkages between paragraphs to increase coherence. We defined Self-Determination Theory (SDT) in full and eliminated use of acronym SDT. We revisited the literature review and reduced unnecessary citations. We have described the research gap that supports our hypotheses.

4. Hypotheses

We have changed the heading to *Study Hypotheses*. We have included a discussion of the three types of time-efficiency in the Literature Review to offer context for the hypothesis. We changed the format of figure references in the hypothesis to (*see Figure*).

5. Methods

We updated the methodology section to better match the style of a manuscript. We've included details about the estimated population, sample size, and sampling method employed. We've included the source of the instrument used to assess student support.

6. Non-normal nature of data

We have expanded the Data Analysis section to address how we treated the non-normal nature of our data. we detail our shift from covariance-based SEM to Partial Least Squares SEM (PLS-SEM) due to its ability to accommodate non-normal data distributions.

7. Data Analysis write-up

We have substantially revised and condensed the Data Analysis section to better reflect the style of a manuscript, focusing on key methodological decisions and their rationale.

8. Control variables

We have added information about the control variables used in our study. Specifically, we mention that we included 'Gender' and 'Specialization' as control variables and report their effects on student engagement.

9. Results reorganization

We have thoroughly revised the Results section to ensure clear alignment between the text and the tables. We have corrected discrepancies, such as the one you pointed out regarding the path coefficient and p-value for the relationship between long-term planning and student engagement. We now ensure that all results mentioned in the text are accurately reflected in the corresponding tables.

10. Discussion revision

We have revised the Discussion section to better suit the style of a manuscript, focusing on interpreting our findings in the context of existing literature and theoretical frameworks.

11. References

We decreased the number of references. We edited the in-text citations and references to meet the journal's standards.

**Reviewer 2**

1. Short-term planning significance

We have expanded our discussion on why short-term planning was found to be less significant in our study. We now offer potential explanations for this unexpected finding and suggest areas for future research to further explore this relationship in e-learning contexts.

2. We have offered more specific definitions of the sorts of student support evaluated in our study. We built on the Saudi Arabian context for e-learning to better explain our findings. We've given examples of challenges students encountered while e-learning. We have included citations to back up the comments about time-efficiency. We have included paragraph at the end of literature review section to demonstrate how our study fills identified gaps. We've added a table that summarizes the participants' demographics. We have offered a more extensive explanation for the decreased importance of short-term planning in our findings
3. Abbreviations

We have spelled out Self-Determination Theory (SDT) and Engagement Theory (ET) and used the full terms more frequently throughout the manuscript.

We believe these revisions have significantly improved the clarity, organization, and overall quality of our manuscript. We appreciate your valuable input and hope that our responses and revisions adequately address your concerns.
