# Supplementary material for: Time efficiency as a mediator between institutional support and higher education student engagement during e-learning
Source: PLoS One. 2025 Jan 24;20(1):e0315420. doi: 10.1371/journal.pone.0315420 (PMC11760031; doi:10.1371/journal.pone.0315420)
Supplement: Attachment — Submitted filename: responses to reviewer 3-round 2.docx [file pone.0315420.s002.docx]

Dear Editors and reviewers.

Thank you very much for your helpful feedback. We appreciate your time and efforts. We are appending a revised manuscript (round 2) entitled “**Appreciative Inquiry in implementing Artificial Intelligence for ELT”**. In the attachments, you will find the revised manuscript. You will find below a point-by-point response to the reviewers’ comments. We have addressed all the comments and agreed with all your valuable suggestions. Thank you again for your cooperation. We hope that the revised manuscript will eventually be accepted by the reviewers and editors.
Sincerely Yours**,**

**Authors**

| **Questions for general evaluation** | **Reviewer’s evaluation** | **Authors’ response** |
| --- | --- | --- |
| Is the content succinctly described and contextualized with respect to previous and present theoretical background and empirical research (if applicable) on the topic? | Must be improved | - The authors modified the findings of their study, appearing in the Discussion and Conclusion sections, to ensure they are well-linked to other studies cited in the literature. Also, they emphasised the value of the appreciative inquiry framework in the Discussion section and conclusion, kindly check the modified sections below:  1- Discussion   “This emphasis on the positivity of AI tools in relation to teaching practices strengthens the appropriateness of the appreciative inquiry approach used to examine positive teaching practices in the field of ELT. The four phases of this approach (i.e., Discovery, Dream, Design and Delivery) mapped the participants’ practices clearly. Following these phases, various positive practices have been identified, such as student teachers’ confidence, learning independency and improved planning. This supports the claim that the appreciative inquiry approach can lead to professional development [6].”   2- Conclusion:  - “These findings contribute to the benefits of AI tools in available literature [17] [28]. However, as confirmed in previous studies [3] [5] [28] [31], concerns of using AI tools were also highlighted in this study,…” - “The researchers hope that this study will enrich research-informed practices for sustainable professional development, and that the reported AI tools can go on to be tested by ELT teachers... ELT researchers are encouraged to implement the appreciative inquiry approach for investigating the practices of ELT teachers to encourage teachers’ new practices and confidence in ELT classrooms [6]. Finally, the findings of this study can inform educational fields other than ELT, since it provides an evaluation of AI tools as implemented by teachers. This may inform the instructional designs of other disciplines and contribute to the professional development of teachers.” |
| Are the research design, questions, hypotheses and methods clearly stated? | Must be improved | - The first research question was paraphrased to specify the target participants:  1. “How do the student teachers perceive the implementation of AI tools for designing their ELT lessons?”  - New details about the tools were added to clarify the purposes of these tools:   “Reflective journals were chosen by the researchers as the main data collection tool because they could provide a detailed account of the participants’ stories in relation to AI tools. In addition to the reflective journals, the participants were asked to write their initial reflections following the first microteaching via a BlackBoard (BB) forum.  The purpose of this BB forum was to build a shared dialogue in which the participants could exchange experiences for their professional development and provoke more initiatives for the implementation of AI tools in their future lesson designs. Thematic analysis was employed for both the reflective journals and BB forum. In order to enhance the study validity, lesson plans were cross-referenced with the four phases of the appreciative inquiry framework, and screenshots of some AI-based activities were inserted (figures 3 and 4).” |
| Are the arguments and discussion of findings coherent, balanced and compelling? | Not applicable | No changes were required. |
| For empirical research, are the results clearly presented? | Not applicable | No changes were required. |
| Is the article adequately referenced? | Must be improved | All the reviewer’s concerns regarding the references were checked and resolved as explained in our response below. |
| Are the conclusions thoroughly supported by the results presented in the article or referenced in secondary literature? | Not applicable | No changes were required. |

**Responses to Reviewer 3**

| **Reviewer’s comments** | **Authors’ response** |
| --- | --- |
| Unfortunately, the authors did not address my comments regarding potential research manipulation in citations and references. The issue of academic research misconduct persists in this revision. For example, on line 117, reference [22] is placed after Reynolds and Taylor. However, [22] corresponds to the work by F. Karataş, F. Y. Abedi, F. Ozek Gunyel, D. Karadeniz, and Y. Kuzgun, titled ‘Incorporating AI in Foreign Language Education: An Investigation into ChatGPT’s Effect on Foreign Language Learners,’ published in *Education and Information Technologies* in March 2024 (doi: 10.1007/s10639-024-12574-6). This suggests that the authors have not adequately addressed citation and referencing, indicating a lack of attention to an aspect that is highly significant to the academic community. | - Thanks for drawing our attention to the reference correct number in the reference list. This is modified now. The reference is identical now in both in-text and reference list. - All in-text citations and reference citations were thoroughly checked by the authors and no errors were identified, apart from the error in line 117 mentioned by the reviewer. - All claims and information stated in the manuscript were double checked for accuracy against the references, and all of these claims and information were accurate. - We appreciate the reviewer’s concerns about the quality of reviewed articles for MDPI, and we would like to ensure the reviewer that the authors are totally aware of research practices and the consequences of research misconduct, and this is reflected in their publication records in SSCI/ SSCE indexed journals. |
